# The rapidly growing burden of dengue fever in China from 2005 to 2024: spatiotemporal patterns and age-sex-specific disparities in incidence and disability-adjusted life years

**DOI:** 10.1186/s40249-026-01516-8

**Published:** 2026-11-10

**Authors:** Sihan Li, Hongrui Zhai, Caihong Xu, Meilan Huang, JunYuan Chen, Qiulan Chen, Yanping Zhang

**Affiliations:** 1 Chinese Center for Disease Control and Prevention (Chinese Academy of Preventive Medicine), Changbai Road, Changping District, Beijing, 102206 China; 2 National Key Laboratory of Intelligent Tracking and Forecasting for Infectious Diseases, Beijing, China; 3 Department of Acute Infectious Diseases, Qingdao Municipal Center for Disease Control and Prevention, Qingdao, China; 4 Department of Epidemiology, School of Public Health, Nanjing Medical University, Nanjing, China; 5 School of Public Health, Guangxi Medical University, Nanning, China

**Keywords:** Dengue fever, Epidemiology, Disease burden, Disability-adjusted life years, Incidence, China

## Abstract

**Background:**

Dengue fever has transitioned from a sporadic imported infection to an established domestic threat in China, challenging the conventional view of its non-endemic status. Understanding its evolving epidemiological landscape over the past two decades is critical for evidence-based intervention planning.

**Methods:**

We analyzed national notifiable dengue surveillance data from 2005 to 2024. Temporal trends were assessed using Joinpoint regression to estimate average annual percent changes (AAPC) in incidence, stratified by age, sex, and province, with particular focus on Guangdong and Yunnan. Spatial heterogeneity was characterized through standardized provincial incidence mapping. Disease burden was quantified using disability-adjusted life years (DALYs), incorporating years of life lost and years lived with disability, based on locally derived severity distributions and disability weights.

**Results:**

A total of 138,885 cases and 14 deaths were reported. Annual case counts rose sharply from 40 to 24,278, with major epidemic peaks in 2014, 2019, 2023, and 2024. The age-standardized incidence increased significantly (AAPC: + 30.1%). Adults aged 25–49 years constituted 49.2% of all cases, yet the steepest relative increases occurred among children aged 0–9 years (male AAPC + 32.7%, female + 35.4%) and adults aged ≥ 70 years (male + 43.3%, female + 39.6%). High-risk category I regions accounted for 87.1% of the national burden, though other low-risk regions exhibited accelerating incidence among specific adult male subgroups. Total DALYs reached 2206.9 person-years (age-standardized rate: 0.0744 per million), predominantly driven by YLD and concentrated in the working-age population (79.5%).

**Conclusions:**

Dengue in China has entered a phase of rapid burden escalation with widening demographic and geographic reach. While the working-age population bears the largest absolute burden, children and older adults face the fastest-rising risk. These findings underscore the urgency of transitioning from reactive outbreak response to precision public health, integrating age-targeted interventions, regionally stratified surveillance, and serological monitoring within a One Health framework to inform future vaccine deployment.

**Supplementary Information:**

The online version contains supplementary material available at https://doi.org/10.1186/s40249-026-01516-8.

## Background

Dengue fever (DF) is an acute vector-borne disease caused by the dengue virus and is primarily transmitted by *Aedes aegypti* and *Aedes albopictus* mosquitoes. It is one of the fastest-spreading mosquito-borne diseases worldwide [1]. In 2019, the World Health Organization (WHO) identified dengue as one of the top ten global public health threats [2]. Infected individuals exhibit a wide spectrum of clinical manifestations, ranging from asymptomatic infection and mild febrile illness (DF) to life-threatening dengue hemorrhagic fever (DHF) and dengue shock syndrome (DSS) [3]. These severe forms are characterized by plasma leakage, severe hemorrhage, organ failure, and shock and are associated with a high case-fatality rate.

Globally, the incidence of DF has increased markedly in recent decades. The number of reported cases worldwide rose from 500,000 in 2000 to 5.2 million in 2019 [4]. In 2023, reported cases exceeded 5 million, with more than 5000 deaths; in 2024, the global outbreak intensified substantially, with reported cases surpassing 14.4 million and deaths exceeding 11,000 [5]. Endemic areas have progressively expanded from traditional tropical and subtropical regions into temperate zones [6], placing over half of the world’s population at risk [7]. Future projections suggest that dengue transmission will continue to intensify, with an increasingly significant public health impact. In China, dengue cases have historically been predominantly imported, primarily originating from Southeast Asian countries [8]. The 20-year period from 2005 to 2024 represents a critical phase of rapid socioeconomic development, accelerated urbanization, and increased international exchange in China. Concurrently, these changes have posed substantial challenges for dengue prevention and control. Major outbreaks occurred in 2014 and 2019. Furthermore, in the post-coronavirus disease 2019 (COVID-19) pandemic years of 2023 and 2024, case numbers surged again. The geographic distribution of dengue has gradually expanded from traditionally affected provinces, such as Guangdong, Fujian, and Yunnan, toward central and northern regions of China [9].

Vector control measures and vaccine development have been the primary prevention strategies, as no specific antiviral treatment for dengue is currently available [10]. To date, two vaccines—CYD-TDV and TAK-003—have been licensed. The WHO recommends the conditional use of CYD-TDV only for individuals with a confirmed prior dengue infection, as this vaccine is effective exclusively in this population and may increase the risk of severe disease upon subsequent infection in dengue-naïve individuals [11]. The 2024 WHO position paper on dengue vaccines recommends integrating TAK-003 into routine immunization programs in high-transmission settings, with priority given to children aged 6–16 years [12]. Therefore, analyzing incidence trends across different age groups and assessing the disease burden within these populations are essential.

The disease burden is commonly quantified using metrics such as disability-adjusted life years (DALYs). This indicator combines years of life lost (YLL) due to premature mortality and years lived with disability (YLD), thereby providing a comprehensive measure of the health loss attributable to a disease [13]. Estimating the DALYs attributable to DF from 2005 to 2024 allows for a comprehensive assessment of its burden in China during this period. This study aims to systematically analyze dengue incidence data in China from 2005 to 2024. By examining long-term incidence trends and their distribution across age groups, the study seeks to characterize the dynamic changes in the dengue epidemic in China and to quantify the epidemiological burden imposed by dengue during this timeframe. These findings are intended to inform dengue prevention and control strategies and to guide future vaccine deployment.

## Methods

### Data sources

DF incidence and mortality data were obtained from the Chinese National Notifiable Infectious Diseases Reporting Information System (NIDRIS). This system serves as the official nationwide platform for communicable disease surveillance in China. Initially established in the 1950s, the system has been progressively strengthened, with major enhancements implemented following the 2003 SARS epidemic. A web-based version of NIDRIS was launched nationwide on January 1, 2004, enabling real-time online reporting of individual cases. This advancement represented a pivotal milestone in China’s infectious disease surveillance infrastructure. DF has been listed as a notifiable disease in China since 1989. The operational structure and reporting procedures of NIDRIS have been described in detail in previous publications [14].

These data included reported case counts and deaths from 31 provinces, autonomous regions, municipalities, and the Xinjiang Production and Construction Corps, and not included cases from Hong Kong, Macao, and Taiwan. Individual-level case data included sex, age, case classification, date of onset, date of diagnosis, and date of death. Dengue cases were not differentiated by source of infection; imported and locally acquired cases were analyzed together. Population data were obtained from the China Center for Disease Control and Prevention Information System, the 2020 Seventh National Population Census, and the Global Burden of Disease (GBD) 2019 standard population weights [15].

### Case definition

DF cases reported from 2005 to 2024 were classified according to the national diagnostic criteria in effect during the corresponding periods: the 2001 criteria (applicable from 2005 to 2008), the 2008 revision (applicable from 2009 to 2017), and the 2018 revision (applicable from 2018 to 2023). Although the overall three-tier diagnostic framework—suspected, clinically diagnosed, and confirmed—remained consistent, the specific case definitions were refined over time. Major revisions included the stricter incorporation of epidemiological exposure in defining suspected cases, the inclusion of both immunoglobulin G and immunoglobulin M seropositivity for clinical diagnosis beginning in 2008, and the formal incorporation of dengue virus nonstructural protein 1 (DENV NS1) antigen detection as a diagnostic criterion in 2018. Laboratory confirmation of confirmed cases—primarily through nucleic acid testing—remained unchanged throughout the study period, thereby ensuring the comparability of confirmed case data across years. A detailed comparison of the diagnostic criteria is provided in Supplementary Table S1.

### Classification of DF Category IV areas

The classification of the four regional categories for DF was defined according to the *Dengue Fever Prevention and Control Protocol (2025 Edition)* [16]. Based on a comprehensive assessment of *Aedes* mosquito distribution patterns and the duration of vector activity, as well as the historical occurrence and scale of local epidemics, the 31 provincial-level administrative divisions (PLADs) directly under the central government and the Xinjiang Production and Construction Corps were categorized into Category I, II, III, and IV regions according to dengue transmission risk, ranked from high to low.

Category I regions (*n* = 6) refer to PLADs with prolonged *Aedes* mosquito activity periods, a higher number of previously reported local cases, and relatively elevated risks of clustered outbreaks. These regions include Zhejiang, Fujian, Guangdong, Guangxi, Hainan, and Yunnan.

Category II regions (*n* = 11) refer to PLADs with relatively prolonged *Aedes* mosquito activity periods or prior reports of local cases, posing a moderate risk of clustered outbreaks. These regions include Shanghai, Jiangsu, Anhui, Jiangxi, Shandong, Henan, Hubei, Hunan, Chongqing, Sichuan, and Guizhou.

Category III regions (*n* = 8) refer to PLADs where *Aedes* mosquitoes are present but no local cases have previously been reported; however, there remains a risk of local transmission due to imported cases. These regions include Beijing, Tianjin, Hebei, Shanxi, Liaoning, Xizang, Shaanxi, and Gansu.

Category IV regions (*n* = 7) refer to areas where no *Aedes* mosquito distribution has been detected and no local cases have previously been reported. These regions include Inner Mongolia, Jilin, Heilongjiang, Qinghai, Ningxia, Xinjiang, and the Xinjiang Production and Construction Corps.

### Calculation of age-standardized rates

The age-standardized rate (ASR) was calculated using the following formula:

$${\mathrm{ASR}} = \Sigma ({{a}}_{{{i}}} \times w_{i} )$$

where ${a}_{i}$ represents the crude rate in the *i*th age group of the study population and ${w}_{i}$ represents the corresponding weight of the *i*th age group in the standard population. Using population data from the 2020 Seventh National Population Census of China as the standard population, the age-standardized incidence rate (ASIR) for each PLAD from 2005 to 2024 was calculated to compare regional differences in dengue incidence across China. The age-standardized DALY rate (ASDR) was calculated using the global standard population weights from the GBD study [15]. Because the age-standardized DALY rates were relatively small in magnitude, all ASDRs are presented per million person-years for clarity.

### Joinpoint regression model

The Joinpoint regression model establishes a segmented regression framework based on the temporal characteristics of disease distribution. It divides the study period into multiple intervals defined by several joinpoints, performs trend fitting and optimization within each segment, and thereby provides a more refined evaluation of segment-specific trends over the overall study period [17]. In this study, Joinpoint (v5.4.0) was used for model construction and analysis, employing a log-linear regression equation as follows:

$$E[y|x]={e}^{{\beta}_{0}+{\beta}_{1}x+{\delta}_{1}{(x-{\tau}_{1})}^{+}+\dots +{\delta}_{k}{(x-{\tau}_{k})}^{+}}$$

In this equation, *y* represents the incidence rate; *x* denotes the observation year; e is the base of the natural logarithm; *k* indicates the number of joinpoints; ${\tau}_{k}$ represents the unknown joinpoints; ${\beta}_{0}$ is the intercept; ${\beta}_{1}$ is the regression coefficient for time; and ${\delta}_{k}$ denotes the regression coefficient corresponding to the *k*-th segment function [16]. The average annual percent change (AAPC) was used to evaluate the overall trend in incidence rates. A positive AAPC indicates an increasing trend, whereas a negative AAPC indicates a decreasing trend. A *P* value < 0.05 was considered statistically significant. The AAPC was calculated using the following formula:

$$AAPC=({e}^{\sum {w}_{i}{\beta}_{i}/\sum {w}_{i}}-1)\times 100$$

In this equation, ${w}_{i}$ represents the interval width of each segment (i.e., the number of years included in that interval), and ${\beta}_{i}$ denotes the regression coefficient corresponding to each segment [17].

In the Joinpoint regression analysis, the dependent variable was the incidence rate, and the independent variable was calendar year. The model was stratified by age group and sex. Trends in incidence were analyzed by sex, age group, and region using crude incidence rates, whereas analyses across all age groups were based on age-standardized incidence rates. The maximum number of joinpoints permitted in the model was set to three, with a minimum of zero joinpoints, and at least two observations were required before, after, and between joinpoints. The error structure was assumed to be uncorrelated, and weighted standard errors were applied. For years with zero reported cases, a continuity correction of 0.0001 was added to the case count for that year. The Joinpoint model was used to calculate crude incidence rates and to reanalyze incidence trends in order to avoid undefined values in the log-linear model. Confidence intervals (*CI*s) were estimated using the empirical quantile method based on 5001 bootstrap resamples. Model selection was performed using permutation testing (maximum of 4499 permutations; α = 0.05).

### Estimation of DALYs

In this study, the simplified DALY formula recommended by the WHO was used to estimate the disease burden. DALYs comprise both YLL due to premature mortality and YLD [18] and are expressed as: The formulas used to calculate YLL and YLD are as follows:

$${\mathrm{YLL}} = {\mathrm{N}} \times {\mathrm{L}}$$

$${\mathrm{YLD}} = {\mathrm{I}} \times {\mathrm{DW}} \times {\mathrm{d}}$$

In these equations, N represents the number of deaths, and L denotes the standard life expectancy at the age of death, derived from the age-specific global life table of the GBD study. This study adopted the Theoretical Minimum Risk Life Table from the GBD 2019 study as the standard reference life table for calculating YLL [15]. This life table is constructed based on the lowest age-specific mortality levels observed globally and assumes a gender-neutral life expectancy at birth of 88.87 years. Values are assigned using the median residual life expectancy corresponding to each age group. In the YLD formula, I represents the number of incident cases, and DW enotes the disability weight, ranging from 0 (full health) to 1 (death). Disability weights were specified separately for adults and children and varied according to disease severity, based on Chinese clinical data [19, 20]. Specifically, for DF cases among children aged < 15 years, the disability weight was set at 0.4160 [19]; severe dengue (SD) cases were assigned a disability weight of 0.6000 [19], with a severe case proportion of 0.5% [20]. For DF cases among individuals aged ≥ 15 years, the disability weight was set at 0.3142 [19]; SD cases were assigned a disability weight of 0.7214 [19], with a severe case proportion of 0.9% [20]. The parameter d represents the average duration of illness. According to published literature, the duration of nonfatal dengue cases typically ranges from 9.5 to 20 days [21]. Setting the duration at 14 days is consistent with the average disease course specified in China’s *Dengue Fever Diagnosis and Treatment Protocol (2024 Edition)* and with other domestic studies that have adopted 14 days as the average duration when calculating DALYs [22, 23]. Based on these considerations, this study defined d as 14 days for DALY estimation, with conversion to years applied during calculation.

### Statistical analysis

A descriptive analysis was conducted on the number of dengue cases and incidence rates in China from 2005 to 2024, stratified by age and sex. Microsoft Excel 2019 (Microsoft Corporation, Redmond, WA, USA) was used to collate and summarize dengue incidence and mortality data, and statistical analyses were performed by year, sex, and age group. A choropleth map illustrating the provincial distribution of age-standardized incidence rates was generated using ArcGIS 10.7 (Environmental Systems Research Institute, Redlands, CA, USA) to explore the spatiotemporal distribution of dengue over the 20-year study period. Joinpoint regression analysis was conducted using R (v4.3.3; R Foundation for Statistical Computing, Vienna, Austria) and Microsoft Excel 2019 (Microsoft Corporation, Redmond, WA, USA) for data processing, with Joinpoint (v5.4.0; IMS, Inc., under contract with the National Cancer Institute, USA) used for model construction and analysis. Incidence trends were evaluated by sex and age group at the national level, and trends in key PLADs were analyzed to estimate the AAPC and its 95% *CI*s, thereby providing evidence to inform adjustments to prevention and control strategies in high-priority PLADs. Using R (v4.3.3), YLL, YLD, and total DALYs attributable to DF in China from 2005 to 2024 were calculated and described by sex, age group, and four regional categories to comprehensively assess the epidemiological burden of the disease.

## Results

### Basic characteristics

From 2005 to 2024, a total of 138,885 new dengue cases were reported across the 31 PLADs directly under the central government and the Xinjiang Production and Construction Corps. The number of reported cases increased from 40 in 2005 to 24,278 in 2024, with the highest annual total of 46,864 recorded in 2014. Two additional peaks occurred in 2019 and 2023, with 22,188 and 19,541 cases reported, respectively. Overall, 74,224 cases (53.4%) occurred in males and 64,661 cases (46.6%) in females. A total of 14 deaths were reported, of which 11 occurred in Guangdong Province (six in 2014, two in 2017, and three in 2019).

### Epidemiological characteristics of DF in China, 2005–2024

#### Age-specific number of DF cases in China, 2005–2024

**Fig. 1:**
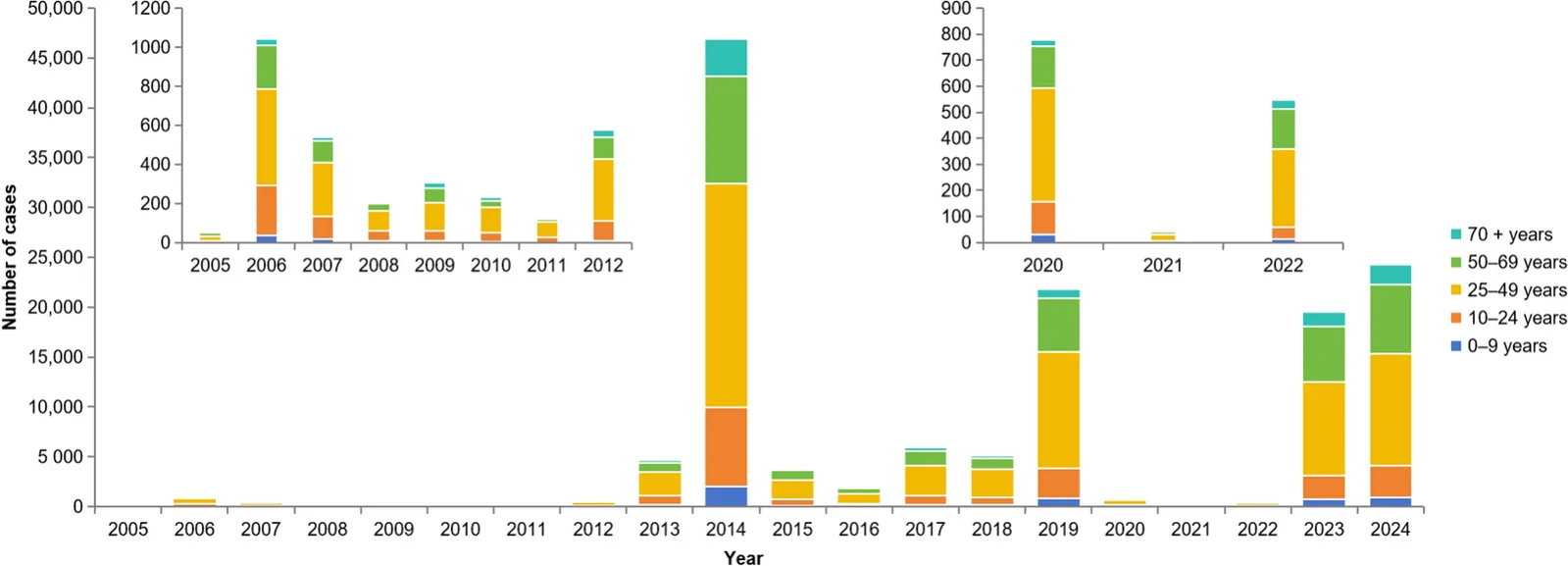
Age-specific number of dengue fever cases in China, 2005–2024

Between 2005 and 2024, the annual number of dengue cases ranged from 40 to 46,864, peaking in 2014, with marked increases observed across all age groups. The proportion of cases in the 25–49-year age group ranged from 46.0% to 66.7%, whereas the 50–69-year age group accounted for 12.5% to 28.8% of cases. The 0–9-year, 10–24-year, and ≥ 70-year age groups consistently represented a smaller proportion of total cases (Fig. 1). From 2005 to 2012, case numbers remained low across all age groups, with the 25–49-year age group accounting for 51.6% of cases. Between 2015 and 2018, the total number of reported cases increased sharply to 16,937, compared with only 3048 during 2005–2012; of these, 8792 cases (51.9%) occurred in the 25–49-year age group. Major outbreaks in 2014, 2019, 2023, and 2024 resulted in substantial increases in case numbers across all age groups. In 2014, the peak outbreak year, 22,475 cases (48.0%) were reported in the 25–49-year age group and 10,789 cases (23.0%) in the 50–69-year age group (Fig. 1).

#### Sex-specific number of cases and incidence rates of DF in China, 2005–2024

**Fig. 2:**
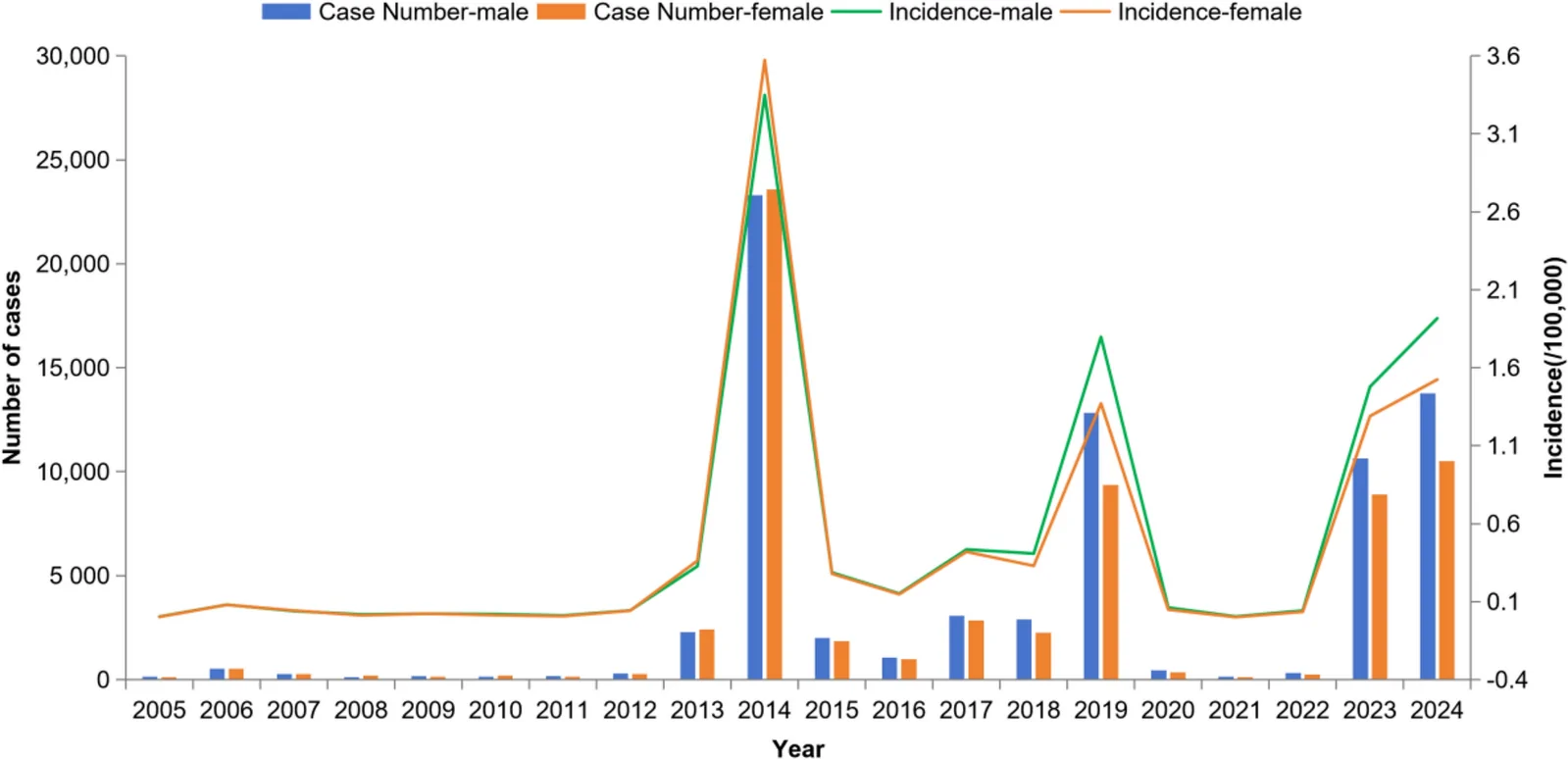
Number of cases and incidence rate by sex for dengue fever in China, 2005–2024

Between 2005 and 2024, the total number of reported dengue cases was higher in males than in females (74,224 vs. 64,661 cases). The reported incidence rate among males increased from 0.0039 per 100,000 population in 2005 to 1.9169 per 100,000 in 2024, whereas the incidence rate among females rose from 0.0022 per 100,000 in 2005 to 1.5244 per 100,000 in 2024. The incidence trends for males and females were generally similar from 2005 to 2018. Notably, in 2019, 2023, and 2024, both the number of cases and the incidence rates remained higher in males than in females (Fig. 2).

#### Provincial variations in dengue fever epidemiological characteristics in China, 2005–2024

The ASIR of DF in China increased from 0.0036 per 100,000 in 2005 to 1.6984 per 100,000 in 2024, with the highest rate of 4.1092 per 100,000 recorded in 2014. The AAPC was + 30.1% (95% *CI*: 20.6–135.5, *P* = 0.008), indicating a statistically significant upward trend. The geographic distribution of dengue expanded from 31 districts/counties across 20 prefecture-level cities in 10 PLADs in 2005 to 895 districts/counties across 217 prefecture-level cities in 28 PLADs by 2024.

**Fig. 3:**
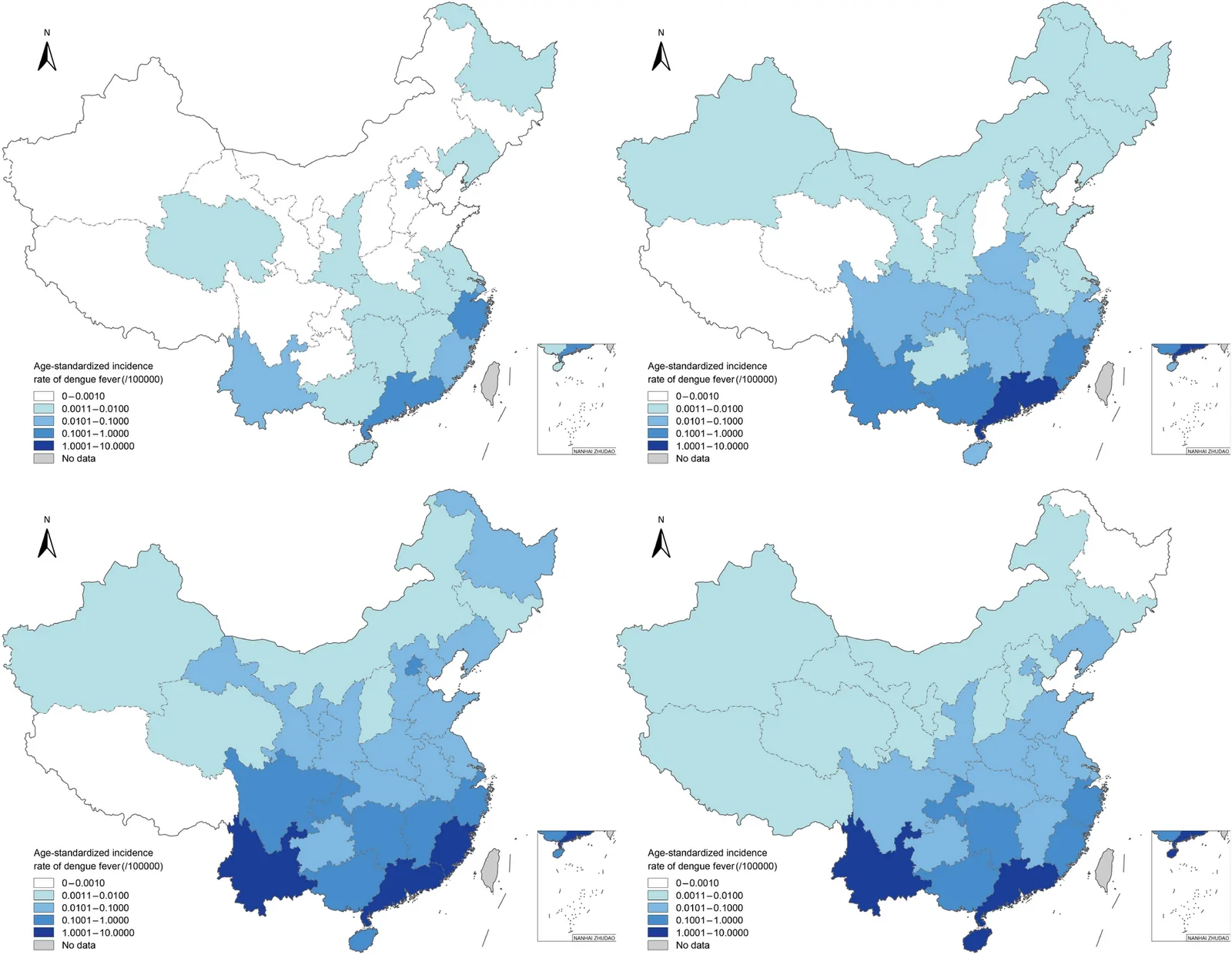
Age-standardized incidence rate of dengue fever by province in China, 2005–2024 (A: 2005–2009; B: 2010–2014; C: 2015–2019; D: 2020–2024). Map approval number: GS(2026)3430

From 2005 to 2009, the ASIR remained relatively low, with a mean of 0.0451 per 100,000 population. Only two provinces, Guangdong and Zhejiang, reported average annual incidence rates exceeding 0.1 per 100,000, with Guangdong recording the highest rate at 0.3857 per 100,000 (Fig. 3; Supplementary Table S2). During 2010–2014, the mean ASIR increased to 0.9145 per 100,000. Guangdong Province recorded a mean rate of 9.6292 per 100,000, while Guangxi, Yunnan, and Fujian also reported average annual incidence rates above 0.1 per 100,000 (Fig. 3; Supplementary Table S2). Between 2015 and 2019, the mean ASIR was 0.5974 per 100,000. Three PLADs—Guangdong (2.4762 per 100,000), Yunnan (5.0298 per 100,000), and Fujian (1.5126 per 100,000)—reported average annual incidence rates exceeding 1.0 per 100,000. Nine PLADs—Guangxi, Hainan, Zhejiang, Chongqing, Jiangxi, Hunan, Sichuan, Shanghai, and Beijing—recorded average annual incidence rates ranging from 0.1 to 1.0 per 100,000 (Fig. 3; Supplementary Table S2). During 2020–2024, the mean ASIR was 0.6342 per 100,000. Three PLADs—Guangdong, Yunnan, and Hainan—reported average annual incidence rates exceeding 1.0 per 100,000, whereas Guangxi, Fujian, Zhejiang, Hunan, and Chongqing had rates ranging from 0.1 to 1.0 per 100,000 (Fig. 3; Supplementary Table S2). From 2005 to 2024, both the geographic distribution and epidemic intensity of dengue in China expanded substantially.

#### Joinpoint regression analysis results

During 2005–2024, the national incidence rate of DF demonstrated a statistically significant upward trend (AAPC: + 30.1%, 95% *CI*: 20.6–135.5, *P* = 0.008), with differences in the magnitude of increase and statistical significance observed across sex and age groups (Table 1). The increasing trend was most pronounced among children and older adults. The overall incidence of dengue fever among females exhibits a statistically significant upward trend (AAPC: + 28.4%, 95% *CI*: 19.3–123.1, *P* = 0.009). The most pronounced increases were observed in the ≥ 70-year age group, with an average annual increase of 39.6% (95% *CI* :28.2–98.1, *P* = 0.007), and in the 0–9-year age group, with an average annual increase of 35.4% (95% *CI*: 24.4–92.2, *P* = 0.012). The overall incidence of dengue fever among males showed a statistically significant upward trend (AAPC: + 31.0%, 95% *CI*: 21.6–139.7, *P* = 0.008), whereas no significant increase was observed in the 25–49 (AAPC: + 4.1%, 95% *CI*: − 2.0–18.3, *P* = 0.105) and 50–69 age groups (AAPC: + 1.3%, 95% *CI*: − 4.6–14.8, *P* = 0.239). The greatest increase occurred in the ≥ 70-year age group, with an average annual increase of 43.3% (95% *CI*: 25.3–134.0, *P* = 0.022), followed by the 0–9-year age group, with an average annual increase of 32.7% (95% *CI*: 20.4–74.1, *P* = 0.010). In several age- and sex-stratified analyses, as well as in analyses of population-wide age-standardized rates, the estimated AAPCs were accompanied by wide 95% *CI*s, reflecting substantial variability, such as 70+ years age group in males (AAPC: + 43.3%, 95% *CI*: 25.3–134.0, *P* = 0.022). This imprecision likely resulted from low case counts, including zero cases in certain years within specific age groups, and marked incidence fluctuations during outbreak years, which reduced the statistical stability of the Joinpoint regression estimates. Although these data limitations contributed to the wide AAPC *CI*s, the upward trend remains epidemiologically meaningful.

**Table 1:** Joinpoint regression analysis of dengue fever incidence trends by sex and age group in China, 2005–2024

Age group	Male			Female		
	ASIR, per 100,000		AAPC (95% *CI*)	ASIR, per 100,000		AAPC (95% *CI*)
	2005	2024		2005	2024	
0–9 years	0	0.7395	+ 32.7^*^(20.4–74.1)	0.0014	0.5708	+ 35.4^*^(24.4–92.2)
10–24 years	0.0023	1.4799	+ 26.4^*^(17.0–93.2)	0.0032	1.1487	+ 27.2^*^(18.1–93.0)
25–49 years	0.0065	2.5866	+ 4.1(− 2.0–18.3)	0.0024	1.8805	+ 28.7^*^(18.5–120.6)
50–69 years	0.0041	1.8610	+ 1.3(− 4.6–14.8)	0.0017	1.6732	+ 27.4^*^(17.3–128.0)
70+ years	0	1.6537	+ 43.3^*^(25.3–134.0)	0	1.4057	+ 39.6^*^(28.2–98.1)
	Age-standardized incidence rate					
All age groups	0.0040	1.9143	+ 31.0^*^(21.6–139.7)	0.0020	1.5231	+ 28.4^*^(19.3–123.1)

*Statistically significant trend (*P* < 0.05); AAPC: average annual percentage change; *CI*: confidence interval; ASIR: age-standardized incidence rate

As a traditionally high-prevalence region, Guangdong Province has consistently recorded the highest incidence levels nationwide, with an AAPC of + 9.7% (95%* CI*: − 12.2 to 29.0, *P* = 0.380); however, this trend did not reach statistical significance (Table 2). In Yunnan Province, incidence rates increased significantly across all sex and age groups, with AAPC values ranging from + 16.6% to + 27.3%, all of which were statistically significant (*P* < 0.001) (Table 2). Among males aged 0–9 years, the incidence rate increased from 0 to 2.2923 per 100,000, with an average annual increase of 27.3%, representing the greatest relative increase in Yunnan Province. Although incidence rates in PLADs other than Guangdong and Yunnan remained relatively low, their growth rates were notably rapid. The overall population AAPC reached + 28.4% (95% *CI*: 21.8–60.8, *P* < 0.001), with the highest increase observed among males aged 50–69 years, whose incidence rate increased by an average of 32.0% per year (Table 2).

**Table 2:** Joinpoint regression analysis of dengue fever incidence trends in Guangdong, Yunnan, and other PLADs in China, 2005–2024

PLAD	Age group	Male			Female		
		ASIR, per 100,000		AAPC (95% *CI*)	ASIR, per 100,000		AAPC (95% *CI*)
		2005	2024		2005	2024	
Guangdong Province	0–9 years	0	5.9710	+ 8.2(− 22.4–28.4)	0	4.6939	+ 9.0(− 16.7–29.3)
	10–24 years	0	12.1287	+ 9.5(− 10.8–24.8)	0	9.8706	+ 10.0(− 10.2–25.4)
	25–49 years	0	15.7025	+ 7.0(− 16.5–26.3)	0.0148	12.5930	+ 6.5(− 13.6–24.8)
	50–69 years	0.0289	17.7551	+ 13.1(− 8.3–34.5)	0.0101	17.4763	+ 11.2(− 10.3–30.3)
	70+ years	0	25.6803	+ 15.4(− 31.6–51.5)	0	20.8974	+ 13.9(− 31.0–41.2)
	Total	Overall population AAPC (95% *CI*)					
		+ 9.7(− 12.2–29.0)					
Yunnan Province	0–9 years	0	2.2923	+ 27.3^*^(17.9–48.4)	0	1.7715	+ 24.1^*^(15.0–45.9)
	10–24 years	0	5.9707	+ 22.8^*^(15.8–43.8)	0	4.3208	+ 18.1^*^(10.5–35.9)
	25–49 years	0.0107	11.5835	+ 26.7^*^(20.9–49.2)	0	9.9909	+ 21.8^*^(14.3–40.1)
	50–69 years	0	9.9622	+ 24.1^*^(18.2–47.0)	0	8.4622	+ 21.1^*^(13.8–40.8)
	70+ years	0	5.6277	+ 16.6^*^(6.5–35.9)	0	5.4790	+ 21.5^*^(10.9–44.6)
	Total	Overall population AAPC (95% *CI*)					
		+ 23.8^*^(17.9–44.6)					
Other PLADs	0–9 years	0	0.0450	+ 28.6^*^(19.2–59.7)	0.0016	0.0311	+ 21.4^*^(10.6–46.1)
	10–24 years	0.0027	0.1621	+ 29.3^*^(21.1–64.8)	0.0036	0.1255	+ 23.0^*^(15.3–49.2)
	25–49 years	0.0068	0.4310	+ 31.8^*^(24.9–74.2)	0.0018	0.2476	+ 25.7^*^(19.3–57.8)
	50–69 years	0.0019	0.2990	+ 32.0^*^(26.6–78.9)	0.0010	0.2094	+ 20.9^*^(15.2–46.9)
	70+ years	0	0.1174	+ 15.7^*^(7.9–37.9)	0	0.0916	+ 15.0^*^(6.8–36.6)
	Total	Overall population AAPC (95% *CI*)					
		+ 28.4^*^(21.8–60.8)					

*Statistically significant trend (*P* < 0.05); AAPC: average annual percentage change;* CI*: confidence interval; PLAD: provincial-level administrative division; ASIR: age-standardized incidence rate

### YLL, YLD, and DALYs for DF in China, 2005–2024

From 2005 to 2024, the total DALYs attributable to DF in China were 2206.9 person-years, with an annual mean of 110.3 person-years (Table 3). The ASDR was 0.0744 per million population (Table 4). YLD constituted the primary component of the total disease burden, accounting for 78.3% (1727.9 person-years) of total DALYs, whereas YLL accounted for 21.7% (479.0 person-years) (Supplementary Table S3). The years 2014, 2019, and 2023 marked peaks in the dengue-related disease burden in China. The highest DALY value was recorded in 2014 at 721.4 person-years, of which YLD (583.7 person-years) accounted for 80.9% (Supplementary Table S3). The DALYs in 2019, 2023, and 2024 were 384.3, 283.1, and 302.0 person-years, respectively. In other years, the disease burden remained comparatively low, particularly during 2006–2013 and 2020–2021.

**Table 3:** DALYs for dengue fever in the four categorical regions of China, 2005–2024

Year	DALY (person-years)				
	Category I regions	Category II regions	Category III regions	Category IV regions	Total
2005	0.3	66.2	0.1	0.0	66.6
2006	12.8	0.2	0.1	0.0	13.1
2007	6.5	0.1	0.1	0.0	6.8
2008	2.2	0.3	0.1	0.0	2.5
2009	3.5	0.2	0.1	0.0	3.8
2010	2.3	0.3	0.1	0.0	2.7
2011	1.1	0.3	0.1	0.0	1.5
2012	6.5	0.4	0.2	0.0	7.1
2013	56.0	1.4	0.5	0.1	57.9
2014	718.0	2.9	0.4	0.1	721.4
2015	45.3	1.9	0.5	0.0	47.7
2016	23.7	1.3	0.4	0.0	25.5
2017	132.8	3.6	0.6	0.1	137.0
2018	57.3	68.9	0.7	0.1	127.0
2019	321.6	59.7	2.5	0.5	384.3
2020	9.0	0.6	0.0	0.0	9.7
2021	0.4	0.0	0.0	0.0	0.5
2022	6.6	0.1	0.0	0.0	6.7
2023	232.3	49.7	0.9	0.2	283.1
2024	283.1	17.9	0.9	0.1	302.0
Total	1921.5	275.9	8.3	1.2	2206.9

DALY: disability-adjusted life year

**Table 4:** Sex-specific DALYs for dengue fever in China, 2005–2024

Year	DALY, person-years			ASDR, per million person-years		
	Male	Female	Total	Male	Female	Total
2005	66.4	0.2	66.6	0.0979	0.0003	0.0506
2006	6.7	6.4	13.1	0.0097	0.0095	0.0096
2007	3.4	3.4	6.8	0.0048	0.0051	0.0050
2008	1.5	1.0	2.5	0.0022	0.0015	0.0019
2009	1.9	1.9	3.8	0.0028	0.0028	0.0028
2010	1.7	1.1	2.7	0.0024	0.0014	0.0019
2011	0.9	0.6	1.5	0.0012	0.0008	0.0010
2012	3.7	3.4	7.1	0.0051	0.0047	0.0049
2013	28.3	29.7	57.9	0.0391	0.0421	0.0405
2014	347.8	373.5	721.4	0.5058	0.5267	0.5160
2015	24.8	22.9	47.7	0.0340	0.0326	0.0333
2016	13.3	12.2	25.5	0.0185	0.0172	0.0179
2017	79.7	57.3	137.0	0.0889	0.0819	0.0855
2018	36.1	91.0	127.0	0.0491	0.1254	0.0865
2019	224.5	159.8	384.3	0.2850	0.2012	0.2440
2020	5.6	4.1	9.7	0.0077	0.0057	0.0067
2021	0.4	0.1	0.5	0.0005	0.0001	0.0004
2022	3.8	3.0	6.7	0.0048	0.0039	0.0044
2023	132.9	150.2	283.1	0.1766	0.1880	0.1822
2024	171.5	130.5	302.0	0.2295	0.1801	0.2053
Total	1154.8	1052.1	2206.9	0.0765	0.0725	0.0744

DALY: disability-adjusted life year; ASDR: age-standardized disability-adjusted life year rate

The total DALYs in Category I regions were 1921.5 person-years, representing 87.1% of the national burden (Table 3). Category II regions accounted for 275.9 person-years (12.5%), whereas Categories III and IV contributed 8.3 person-years (0.4%) and 1.2 person-years (0.1%), respectively (Table 3). The DALYs in Category II regions increased markedly after 2018, peaking at 68.9 person-years in 2018. In contrast, the disease burden in Categories III and IV remained consistently low throughout the study period.

Between 2005 and 2024, the total DALY burden was slightly higher in males (1154.8 person-years) than in females (1052.1 person-years) (Table 4). The ASDR was 0.0765 per million population in males and 0.0725 per million population in females (Table 4). The YLL burden attributable to dengue was higher in females (249.4 person-years) than in males (229.6 person-years), whereas the YLD burden was higher in males (925.1 person-years) than in females (802.7 person-years) (Supplementary Table S3).

Significant disparities in dengue burden were observed across age groups in China. The burden was predominantly concentrated in individuals aged 15–64 years, accounting for 79.5% of total DALYs (Table 5). The 25–34-year age group bore the highest burden, with 430.0 person-years (19.5%), corresponding to an ASR of 0.1078 per million population (Table 5). The disease burden among children was relatively low, with those aged 0–14 years accounting for only 6.6% of total DALYs. Although the total DALYs among older adults aged 75–84 years were low, their ASDR was comparable to that of the 25–34-year age group, at 0.1027 per million population (Table 5). The ASDR was similar across three age groups—45–54 years, 55–64 years, and 65–74 years—with rates of 0.0996, 0.0918, and 0.0969 per million population, respectively. However, the absolute DALY values differed, amounting to 410.1, 278.8, and 206.6 person-years for these age groups (Table 5).

**Table 5:** Age-specific DALYs for dengue fever in China, 2005–2024

Age group	YLL, person-years	YLD, person-years	DALY, person-years	ASDR, per million person-years	Proportion of DALY, %
0–4	0.0	33.5	33.5	0.0223	1.5
5–14	0.0	112.8	112.8	0.0350	5.1
15–24	66.1	206.2	272.4	0.0643	12.3
25–34	63.2	366.9	430.0	0.1078	19.5
35–44	46.4	318.2	364.6	0.0812	16.5
45–54	124.7	285.5	410.1	0.0996	18.6
55–64	65.2	213.6	278.8	0.0918	12.6
65–74	87.0	119.6	206.6	0.0969	9.4
75–84	26.5	59.1	85.6	0.1027	3.9
85+	0.0	12.5	12.5	0.0541	0.6
Total	479.0	1727.9	2206.9	0.0744	100.0

YLL: years of life lost; YLD years lived with disability; DALY disability-adjusted life year; ASDR age-standardized disability-adjusted life year rate

## Discussion

The findings indicate that dengue incidence in China exhibited a statistically significant upward trend from 2005 to 2024, characterized by distinct intermittent outbreak patterns. The 25–49-year age group consistently accounted for the highest number of cases, and the incidence rate was slightly higher in males than in females. The risk of infection increased markedly across the population, with the highest average annual increases observed in the ≥ 70-year and 0–9-year age groups. Significant increases in incidence were also observed in Yunnan Province and in other PLADs outside Guangdong and Yunnan. The disease burden was primarily driven by YLD and was slightly higher in males than in females. The distribution of dengue burden was uneven across age groups, with young and middle-aged adults bearing the largest share. After age standardization, the burden remained particularly high among individuals aged 25–34 years and older adults aged 75–84 years. Geographically, the burden was markedly imbalanced, with Category I regions accounting for 87.1% of the total dengue burden.

DF in China is characterized by intermittent outbreaks, typically with peak incidence occurring every 3–5 years. Between 2005 and 2024, China experienced multiple outbreaks, with two major peaks in 2014 and 2019. In response to the 2014 epidemic, the Chinese Center for Disease Control and Prevention (China CDC) developed and issued several technical guidelines [24], including those addressing DF case surveillance, laboratory testing and diagnosis, vector surveillance, and vector control, thereby establishing a relatively comprehensive national technical framework for dengue prevention and control. Dengue virus non-structural protein 1 (NS1) antigen detection was introduced in China around 2010 and was widely implemented after the 2014 Guangzhou outbreak [25]. In 2018, the national diagnostic criteria for dengue were revised to incorporate NS1 antigen detection as a basis for clinical diagnosis, replace the classifications of DHF and DSS with SD, and introduce warning signs for severe disease [26]. The revised diagnostic criteria were officially implemented in August 2018. The second major outbreak occurred in 2019, which marked the last epidemic year before the COVID-19 pandemic and constituted a natural boundary for subsequent epidemiological patterns. Accordingly, the analysis of provincial variation in DF incidence over the past two decades was conducted across four time periods.

The temporal analysis of dengue incidence from 2005 to 2024 should be interpreted within the context of evolving surveillance systems and diagnostic practices. The stability of the national web-based NIDRIS since 2004 has provided a consistent framework for data collection. Although the diagnostic criteria were revised in 2008 and 2018—most notably through the incorporation of NS1 antigen testing as a basis for clinical diagnosis in 2018—laboratory confirmation, primarily through nucleic acid testing, remained the unchanged gold standard for definitive cases throughout the study period. This consistency ensures the comparability of confirmed case trends. However, major outbreaks in 2014 and 2019 prompted intensified surveillance, enhanced physician training, and the widespread deployment of rapid NS1 tests, which likely improved case detection and reporting, particularly for milder or earlier presentations. Consequently, increases in reported incidence, especially during outbreak years, may reflect a combination of true epidemic activity and improved diagnostic sensitivity, representing a recognized limitation in long-term surveillance analyses.

From a temporal dynamics perspective, the incidence peaks observed in 2014 and 2019 are consistent with previously reported findings in the literature [27]. The marked decline in reported dengue incidence in China between 2020 and 2022 coincided with the implementation of stringent non-pharmacological interventions during the COVID-19 pandemic. A global meta-analysis suggests that such measures—particularly international travel restrictions—may reduce dengue importation in non-endemic settings, whereas restrictions on internal mobility may mitigate domestic transmission [28]. Given that dengue outbreaks in China are frequently initiated by imported cases, border controls and reduced domestic mobility during this period provide a plausible and mechanistically supported explanation for the observed decline. However, other factors—including changes in healthcare-seeking behavior, potential under-ascertainment during periods of healthcare system strain, and sustained vector control activities—may also have contributed to the reduced number of reported cases.

The incidence of DF showed minimal disparity between sexes, with both the number of cases and the incidence rate consistently slightly higher in males than in females. However, the burden of dengue was not uniformly distributed across age groups. This study found that the 25–49-year age group consistently accounted for the highest number of cases, a pattern that differs from that observed in traditionally high-endemic regions, where children typically constitute the majority of cases [29]. In endemic regions such as Southeast Asia, dengue is continuously transmitted, and children, through repeated infections with different serotypes, gradually acquire immunity while also representing the majority of reported cases [30]. In contrast, China experiences intermittent outbreaks primarily triggered by imported cases. The adult population generally lacks immunity acquired through prior exposure. Consequently, when the virus is introduced, the susceptible adult population is at risk of infection, leading to a shift in the age distribution of cases toward older age groups. The higher incidence in the 25–49-year age group may be related to occupational characteristics (e.g., outdoor work and business travel) and daily commuting patterns, which may increase exposure to mosquito bites. The high number of cases in the 50–69-year age group also warrants attention. Individuals in this demographic may have a higher prevalence of underlying conditions (such as hypertension and diabetes), and their risk of progressing to severe disease or death following dengue infection is substantially higher than that of younger groups [31]. Hospitalization duration, incidence of complications, and mortality rates are also markedly higher than those observed in younger patients [32, 33]. Consequently, the disease burden in this age group may be underestimated.

Although the 25–49-year age group reported the highest number of cases, the most rapid increases in incidence rates were observed in the ≥ 70-year and 0–9-year age groups. This accelerating trend in these two age groups warrants heightened attention. Children and older adults are high-risk populations for progression to SD. Children have relatively immature immune systems, whereas older adults often have underlying chronic conditions. Both groups frequently participate in community activities and may have limited awareness of, or capacity for, self-protection. If community mosquito vector density remains inadequately controlled, their risk of infection increases substantially. Furthermore, this study highlights a particularly high disease burden among older adults, underscoring the need to enhance clinical surveillance and strengthen prevention and treatment strategies for severe cases in this vulnerable population. Among males aged 25–49 and 50–69 years, the absolute incidence rate increased during the study period; however, this trend did not reach statistical significance according to the AAPC (*P* > 0.05). This discrepancy may be explained by several factors. First, the rising incidence in these age groups did not follow a monotonic pattern and exhibited interannual variability. Second, the relatively small number of cases within these strata resulted in wider CIs for the AAPC estimates. Third, the Joinpoint regression model may have identified nonlinear patterns characterized by an initial increase followed by a decline, leading the overall AAPC to fail to reach statistical significance. Although the results within these strata were not statistically significant, a non-significant AAPC does not imply the absence of a temporal trend; rather, the available evidence may be insufficient to detect such a trend.

The interpretation of these findings for vaccine policy requires careful consideration of the rapid increase in pediatric incidence in the context of a persistently low absolute disease burden. Current WHO recommendations restrict the use of the TAK-003 vaccine to children aged 6–16 years in settings with high dengue transmission intensity, as indicated by a seroprevalence exceeding 60% by age 9 years (SP9) [12]. The national surveillance data presented in this study, although indicating a concerning upward trend, are insufficient to determine whether specific regions meet this threshold. The alternative vaccine, CYD-TDV, is associated with an increased risk of SD in seronegative individuals [34], rendering it unsuitable for use without prior individual screening and thereby limiting its broader application [10]. In China’s largely seronaive pediatric population, such an approach would require a complex and costly screening program for a currently low absolute risk, making it impractical as a public health strategy at present. Therefore, the implementation of a nationwide age-based vaccination campaign would be premature and inconsistent with the current risk–benefit framework.

A critical and immediate next step is the implementation of targeted, age-stratified serological surveys, with a particular focus on children. These surveys should be designed to achieve three objectives: determine age-specific seroprevalence, including calculation of the SP9 metric across different geographic units (e.g., high-risk townships in Yunnan and Guangdong); map heterogeneity in transmission intensity to identify subnational areas where transmission may already be high and concentrated, thereby qualifying as potential pilot zones for vaccine introduction; and establish a baseline for monitoring temporal changes in transmission dynamics. Data derived from such surveys would shift the vaccine policy discussion from a theoretical national debate to an evidence-based, localized decision-making process, ensuring that any future vaccination strategy is epidemiologically justified and confers a clear net benefit to pediatric populations at increasing risk.

Dengue in China is generally considered to result from local transmission initiated by imported cases [35, 36]. Guangdong and Yunnan Provinces are the primary endemic regions. Previous studies have identified close genetic relationships between viral sequences of locally circulating strains and those detected in Southeast Asian countries [37–39]. Owing to combined factors such as climate change and socioeconomic development, the risk of local dengue transmission has increased. However, there is currently insufficient evidence to conclude that sustained endemic transmission has been established in China. Dengue virus comprises four serotypes, all of which have been reported in China, with DENV-1 and DENV-2 predominating [40, 41]. Since 2011, DENV-1 and DENV-2 have caused annual outbreaks, whereas DENV-3 has been associated with local epidemics in 2013, 2015, 2016, and 2019. DENV-4 has been detected primarily in imported cases, with only a limited number of locally transmitted cases reported [37, 42]. Genotypic recombination and the co-circulation of multiple genotypes have been documented across all serotypes [41]. In recent years, outbreaks in Guangdong and Yunnan Provinces have been particularly severe, posing substantial public health challenges. As a historically high-incidence region, Guangdong Province has developed a relatively mature prevention and control system, including comprehensive surveillance systems, integrated vector management strategies, and established public health emergency response mechanisms. These measures have, to some extent, mitigated the rapid increase in incidence rates. However, this does not imply that control efforts can be relaxed, as local outbreaks remain an ongoing public health threat. The marked rise in incidence in Yunnan Province and other regions underscores persistent and substantial challenges. As a border province, Yunnan faces elevated risks associated with increasing international tourism and trade, internal labor migration, climate change, and the expanding geographic distribution of vector mosquitoes. The 27.3% average annual increase in incidence among males aged 0–9 years highlights the rapid expansion of transmission within this low-immunity population and warrants focused attention. In other provinces, the incidence rate among males aged 50–69 years increased by an average of 32.0% per year, reflecting the general lack of population immunity, whereby imported cases can readily initiate local outbreaks. Given the sustained pressure from case importation, dengue prevention and control strategies require further optimization. Preventing importation and interrupting subsequent local transmission are essential. Continued information exchange with dengue-endemic countries, particularly those in Southeast Asia, should be maintained; port surveillance should be strengthened; and integrated measures—including breeding site elimination and evidence-based vector control in high-risk areas—should be implemented to prevent local transmission initiated by imported cases.

Between 2005 and 2024, dengue in China resulted in a total health loss of 2206.9 person-years, with an ASDR of 0.0744 per million population. The primary component of this burden was YLD. During the study period, dengue caused only 14 deaths, resulting in a very low proportion of YLL. The severity and case-fatality rates of dengue in China are extremely low, largely reflecting the country’s prevention and control measures and rigorous case management. First, adherence to the principles of early detection, early diagnosis, and early treatment, together with prompt identification and management of severe cases, is critical for reducing case-fatality rate. *Diagnosis for dengue fever (WS 216*–*2018)* [26] specify high-risk groups for severe disease and early warning signs, provide guidance on supportive treatment, and mandate mosquito-proof isolation during hospitalization. Second, substantial emphasis is placed on dengue prevention and control. As a Category B notifiable infectious disease, dengue is subject to strict reporting and management requirements. In high-risk regions, screening strategies based on testing all patients presenting with fever facilitate early case detection. However, even non-SD cases are typically characterized by high fever, severe pain, and rash [43], often leading to considerable short-term disability and productivity loss. Secondary infections are more likely than primary infections to be associated with severe clinical manifestations [44]. Although most patients recover, the acute phase is associated with substantial health loss and functional impairment. These findings indicate that focusing solely on mortality would substantially underestimate the socioeconomic and productivity impacts of dengue. The greatest burden arises from short-term incapacitation of the working-age population, generating both direct health losses and indirect economic costs.

Xu et al. [23] applied a global disability weight of 0.81 for dengue to estimate its burden in China; however, this approach may overestimate the true burden, given the notably low rates of severe cases and fatalities observed in China. In contrast, the present study differentiated between children and adults and assigned disability weights according to disease severity. These weights were derived from aggregated analyses of previous studies on dengue-related disability weights in China and are therefore more reflective of the actual national disease burden. Compared with the global dengue burden [45], China’s burden remains relatively low, with lower rates of severe illness and mortality, reflecting substantial investments in epidemic control. Nevertheless, the significant upward trends in incidence and disease burden identified in this study suggest that current control efforts remain insufficient. From 1990 to 2021, the incidence of malaria decreased substantially, and its DALY burden declined markedly [46]. Similarly, the disease burdens of schistosomiasis [47], leishmaniasis [48], and other tropical parasitic diseases have demonstrated sustained downward trends. In contrast, both the incidence and disease burden of dengue have shown a persistent upward trajectory, underscoring the urgent need for more effective integrated control strategies.

China’s effective dengue control strategy reflects the “One Health” approach, implemented through a government-led, multisectoral and multidisciplinary system. This integrated model combines case management, vector surveillance, community mobilization, and health education, achieving coordinated governance across human, animal, and environmental domains to interrupt disease transmission effectively [48, 49]. Meanwhile, climate change, urbanization, and land-use changes are key environmental drivers increasing dengue risk in China [50]. Evidence indicates that rising temperatures have expanded the northern geographic range and prolonged the seasonal activity of Aedes albopictus. Rapid urbanization promotes the proliferation of aquatic breeding sites, and cross-border mobility increases the risk of case importation. Although early serological surveys in Yunnan and Hainan (1980s–1990s) suggested potential animal reservoirs (e.g., monkeys, bats, and pigs), contemporary systematic investigations remain limited [51]. Persistent challenges include fragmented data integration for early warning, increasingly complex urban ecological environments, and insufficient understanding of the ecological role of animal hosts in viral maintenance. Future efforts should prioritize the application of big data, artificial intelligence, and the Internet of Things to develop intelligent early warning systems, strengthen continuous surveillance of potential animal hosts, and enhance cross-sectoral and international collaboration within the One Health framework to mitigate long-term environmental and climatic risks.

This study has several limitations. First, the use of data from a passive surveillance system may lead to underestimation of the true incidence. Dengue frequently presents as mild or asymptomatic infection, with asymptomatic cases accounting for up to 75% of infections; consequently, underreporting is common worldwide [52]. Globally, modeled estimates suggest that only one in 5 to 20 dengue infections are reported [53], and a systematic analysis in Southeast Asia estimated an average of eight actual cases for each reported case [54]. Although a precise national underreporting multiplier has not been established for China, this inherent limitation implies that the reported incidence trends reflect surveillance sensitivity and patterns of severe or medically attended disease. Therefore, the observed increases likely represent a combination of true transmission growth and improvements in diagnosis and reporting. Second, this study applied a uniform severe-case rate across subgroups to estimate DALYs, which may have introduced bias into the results. Future research should conduct systematic investigations of age- and sex-specific severe-case rates of dengue in China to improve the precision of burden estimates. Third, the joinpoint regression analysis in this study was constrained by data sparsity. In age- and sex-stratified analyses with extremely few cases or zero incidence rates across multiple years, the AAPC *CI*s were wide, indicating limited precision and reduced model stability. Future studies may obtain more stable estimates by extending the observation period or aggregating data across multiple years. Additionally, this study did not distinguish between locally acquired and imported cases. Although the epidemiology of dengue in China is characterized by initial introductions from endemic regions followed by local transmission, the reported burden largely reflects the magnitude of established local outbreaks [27]. Available data indicate that locally acquired cases consistently constituted the majority of reported cases in most years, accounting for more than 80% in several years (e.g., 2016, 2018, 2022, and 2024) and exceeding 90% in 2014 and 2023. However, the inability to separate these two sources of cases precludes a more nuanced assessment of the extent to which imported cases initiate local epidemics. Future studies incorporating detailed travel history data are needed to quantify the distinct contributions of imported and indigenous cases to the overall disease burden.

## Conclusions

This twenty-year period (2005–2024) analysis indicates that dengue in China is not only re-emerging but also evolving in its epidemiological characteristics. The disease burden has increased substantially, with a clear shift toward the productive working-age population (25–49 years), which accounted for nearly half of all cases. Alarmingly, the most rapid growth in incidence was observed among the most vulnerable populations—young children and older adults—signaling an expansion of the at-risk population.

The spatial analysis confirms the sustained high burden in Category I provinces (e.g., Guangdong and Yunnan) while highlighting concerning geographic expansion and accelerated growth in previously lower-risk regions. The estimated disease burden, predominantly driven by YLD, further quantifies the substantial socioeconomic impact beyond mortality.

These findings support the direction and necessity of China’s current four-tiered, risk-stratified provincial strategy. This framework moves beyond a one-size-fits-all approach; however, our data highlight opportunities for further refinement within this precision public health paradigm. We recommend reinforcing this stratified strategy with a dual focus.

First, regionally stratified interventions should be strengthened. Although control measures are appropriately intensified in Category I (high-risk) provinces, the observed rapid growth in Category II and III regions underscores the need to proactively enhance surveillance sensitivity and the capacity to contain outbreaks and local transmission. Such measures are essential to prevent endemic establishment and further geographic spread. Sustained vigilance in Category IV regions remains critical for the early detection of imported cases and potential vector presence.

Second, age-targeted protections should be strengthened within each region. Geographic stratification must be complemented by demographic stratification. For the high-burden working-age population, particularly outdoor laborers present across regions, integrating vector control into occupational health programs and delivering targeted education are essential. For the rapidly increasing pediatric and older adult populations—even in lower-category regions—public health efforts should emphasize community-based protection and heightened clinical vigilance. Furthermore, targeted age-stratified serosurveillance in high-risk areas (e.g., Yunnan) is critical to accurately assess transmission intensity and to inform future decisions regarding interventions such as vaccination.

Ultimately, curbing the escalating dengue burden in China requires continuous reinforcement and dynamic adaptation of the integrated “One Health” surveillance and control system. The evolving age-space–time risk patterns identified in this study should serve as a key evidence base for periodically refining the four-category risk classification framework and the corresponding intervention portfolios for each province.

## Supplementary Information

Supplementary material 1.

## Data Availability

The datasets used and/or analysed during the current study are available from the corresponding author on reasonable request.
