# Supplementary material for: The rapidly growing burden of dengue fever in China from 2005 to 2024: spatiotemporal patterns and age-sex-specific disparities in incidence and disability-adjusted life years
Source: Infect Dis Poverty. 2026 Nov 10;15:116. doi: 10.1186/s40249-026-01516-8 (PMC13749878; doi:10.1186/s40249-026-01516-8)
Supplement: Supplementary file 1 [file 40249_2026_1516_MOESM1_ESM.docx]

**Appendix**

**Supplementary Table S1:** Comparative Evolution of Case Definitions for Dengue Fever in China: National Standards (2001, 2008, 2018)

| **Comparison Dimension** | **WS 216-2001 (2001 Edition)** | **WS 216-2008 (2008 Edition)** | **WS 216-2018 (2018 Edition)** | **Summary of Key Evolutions** |
| --- | --- | --- | --- | --- |
| 1. Suspected Case | 1. In endemic area/season: Presence of typical clinical manifestations. 2. In non-endemic area: Presence of typical clinical manifestations and travel history to an endemic area within 15 days prior to symptom onset. | Must incorporate epidemiological link: 1. Epidemiological history and acute febrile illness. 2. Or No clear epidemiological history, but presence of fever, leukopenia, and thrombocytopenia. | Meeting one of the following: a) Epidemiological history and acute febrile illness. b) Acute febrile illness and leukopenia and/or thrombocytopenia. | Centralization of Epidemiology: Since 2008, an epidemiological link became a core element for defining a suspected case, or specific laboratory abnormalities were required, making the criteria more stringent. |
| 2. Clinically Diagnosed Case(probable cases) | A suspected case, and: 1. Positive for DENV-specific IgM antibody (single serum sample). 2. Or ≥4-fold rise in DENV-specific IgG antibody titer between acute and convalescent sera. | Meeting one of the following: 1. A suspected case (pathway 1) with leukopenia and thrombocytopenia. 2. A suspected case (pathway 2) with positive serum IgM or IgG antibody (single sample). 3. Meeting specific criteria for Dengue Hemorrhagic Fever (DHF) or Dengue Shock Syndrome (DSS). | Meeting one of the following: a) A suspected case (pathway a) with rash/hemorrhagic tendency and leukopenia/thrombocytopenia. b) A suspected case with positive serum IgM antibody or NS1 antigen. | Broadening and Innovation of Diagnostic Basis: 1. 2008: Serological criteria expanded from IgM-only to positive for either IgM or IgG (single sample). 2. 2018: Detection of NS1 antigen within 5 days of illness onset was formally included alongside IgM positivity as a core criterion for clinical diagnosis, significantly enhancing early diagnostic capability. |
| 3. Confirmed Case | 1. Isolation of Dengue Virus. 2. ≥4-fold rise in DENV-specific IgG antibody titer. 3. Detection of viral gene sequence by RT-PCR. | A clinically diagnosed case, and meeting one of the following: 1. Virus isolation. 2. ≥4-fold rise in DENV-specific IgG antibody titer. 3. Detection of viral gene sequence by RT-PCR or real-time fluorescence quantitative PCR. | A suspected or clinically diagnosed case, and meeting one of the following: 1. ≥4-fold rise in DENV-specific IgG antibody titer or seroconversion. 2. Virus isolation. 3. Detection of viral nucleic acid by RT-PCR or real-time fluorescence quantitative RT-PCR. | Evolution of Gold Standard Techniques: The role of nucleic acid testing (PCR) was increasingly emphasized. Real-time fluorescence quantitative PCR was added in 2008, and specified as "real-time fluorescence quantitative RT-PCR" in 2018, becoming the mainstream and highly sensitive method for confirmation. |
| 4. Definition of Severe Case | Not listed as a separate diagnostic category. Described as "Dengue Hemorrhagic Fever (DHF)" and "Dengue Shock Syndrome (DSS)" within clinically diagnosed cases. | Not listed as a separate diagnostic category. Diagnostic criteria for DHF and DSS were included within clinically diagnosed cases. | Addition of a separate diagnostic category "Severe Dengue": A clinically diagnosed or confirmed case presenting with one of the following: severe hemorrhage, shock, or severe organ impairment. | Unification and Independence of Classification: The 2018 edition adopted the WHO 2009 guidelines, using "Severe Dengue" to encompass all severe manifestations and elevating it to an independent diagnostic category, emphasizing early identification and management. |

Table S2 The ASIR of dengue fever in different PLADs of China by stage

| Region | PLAD | Annual average ASIR (per 100,000) by province at various stages | | | |
| --- | --- | --- | --- | --- | --- |
|  |  | 2005–2009 | 2010–2014 | 2015–2019 | 2020–2024 |
| Category I regions | Guangdong | 0.3857 | 9.6292 | 2.4762 | 3.4274 |
|  | Guangxi | 0.0055 | 0.3555 | 0.7153 | 0.4441 |
|  | Yunnan | 0.0354 | 0.7702 | 5.0298 | 7.5985 |
|  | Fujian | 0.0957 | 0.2016 | 1.5126 | 0.1787 |
|  | Hainan | 0.0080 | 0.0426 | 0.8728 | 1.0068 |
|  | Zhejiang | 0.1033 | 0.0351 | 0.9136 | 0.2003 |
| Category II regions | Shanghai | 0.0122 | 0.0100 | 0.1295 | 0.0811 |
|  | Jiangsu | 0.0023 | 0.0080 | 0.0782 | 0.0434 |
|  | Anhui | 0.0017 | 0.0031 | 0.0466 | 0.0191 |
|  | Jiangxi | 0.0010 | 0.0119 | 0.5660 | 0.0535 |
|  | Shandong | 0.0000 | 0.0030 | 0.0459 | 0.0150 |
|  | Henan | 0.0004 | 0.0150 | 0.0725 | 0.0254 |
|  | Hubei | 0.0022 | 0.0183 | 0.0957 | 0.0795 |
|  | Hunan | 0.0037 | 0.0282 | 0.2876 | 0.8246 |
|  | Chongqing | 0.0058 | 0.0269 | 0.9880 | 0.1002 |
|  | Sichuan | 0.0002 | 0.0114 | 0.1184 | 0.0703 |
|  | Guizhou | 0.0000 | 0.0022 | 0.0403 | 0.0317 |
| Category III regions | Beijing | 0.0182 | 0.0551 | 0.1127 | 0.0446 |
|  | Tianjin | 0.0000 | 0.0020 | 0.0182 | 0.0102 |
|  | Hebei | 0.0003 | 0.0036 | 0.0224 | 0.0079 |
|  | Shanxi | 0.0005 | 0.0004 | 0.0076 | 0.0065 |
|  | Liaoning | 0.0051 | 0.0048 | 0.0286 | 0.0115 |
|  | Shaanxi | 0.0060 | 0.0074 | 0.0308 | 0.0155 |
|  | Gansu | 0.0005 | 0.0049 | 0.0128 | 0.0050 |
|  | Tibet | 0.0000 | 0.0000 | 0.0000 | 0.0058 |
| Category IV regions | Heilongjiang | 0.0014 | 0.0026 | 0.0145 | 0.0006 |
|  | Jilin | 0.0000 | 0.0020 | 0.0099 | 0.0018 |
|  | Xinjiang | 0.0000 | 0.0021 | 0.0012 | 0.0039 |
|  | Inner Mongolia | 0.0000 | 0.0016 | 0.0020 | 0.0068 |
|  | Ningxia | 0.0000 | 0.0000 | 0.0222 | 0.0055 |
|  | Qinghai | 0.0059 | 0.0000 | 0.0027 | 0.0064 |

PLAD provincial-level administrative division; ASIR age-standardized incidence rate.

Table S3 YLLs, YLDs, and DALYs for dengue fever in China, 2005–2024

| Year | YLL (person-years) | | |  | YLD (person-years) | | |  | DALY (person-years) | | |
| --- | --- | --- | --- | --- | --- | --- | --- | --- | --- | --- | --- |
|  | Male | Female | Total |  | Male | Female | Total |  | Male | Female | Total |
| 2005 | 66.1 | 0.0 | 66.1 |  | 0.3 | 0.2 | 0.5 |  | 66.4 | 0.2 | 66.6 |
| 2006 | 0.0 | 0.0 | 0.0 |  | 6.7 | 6.4 | 13.1 |  | 6.7 | 6.4 | 13.1 |
| 2007 | 0.0 | 0.0 | 0.0 |  | 3.4 | 3.4 | 6.8 |  | 3.4 | 3.4 | 6.8 |
| 2008 | 0.0 | 0.0 | 0.0 |  | 1.5 | 1.0 | 2.5 |  | 1.5 | 1.00 | 2.5 |
| 2009 | 0.0 | 0.0 | 0.0 |  | 1.9 | 1.9 | 3.8 |  | 1.9 | 1.9 | 3.8 |
| 2010 | 0.0 | 0.0 | 0.0 |  | 1.7 | 1.1 | 2.7 |  | 1.7 | 1.0 | 2.7 |
| 2011 | 0.0 | 0.0 | 0.0 |  | 0.9 | 0.6 | 1.5 |  | 0.9 | 0.6 | 1.5 |
| 2012 | 0.0 | 0.0 | 0.0 |  | 3.7 | 3.4 | 7.1 |  | 3.7 | 3.4 | 7.1 |
| 2013 | 0.0 | 0.0 | 0.0 |  | 28.3 | 29.7 | 57.9 |  | 28.3 | 29.7 | 58.0 |
| 2014 | 56.8 | 80.9 | 137.7 |  | 291.1 | 292.6 | 583.7 |  | 347.8 | 373.5 | 721.3 |
| 2015 | 0.0 | 0.0 | 0.0 |  | 24.8 | 22.9 | 47.7 |  | 24.8 | 22.9 | 47.7 |
| 2016 | 0.0 | 0.0 | 0.0 |  | 13.3 | 12.2 | 25.5 |  | 13.3 | 12.2 | 25.5 |
| 2017 | 41.6 | 22.2 | 63.7 |  | 38.1 | 35.2 | 73.3 |  | 79.7 | 57.3 | 137.0 |
| 2018 | 0.0 | 63.2 | 63.2 |  | 36.1 | 27.8 | 63.9 |  | 36.1 | 91.0 | 127.1 |
| 2019 | 65.2 | 43.5 | 108.6 |  | 159.3 | 116.3 | 275.6 |  | 224.5 | 159.8 | 384.3 |
| 2020 | 0.0 | 0.0 | 0.0 |  | 5.6 | 4.1 | 9.7 |  | 5.6 | 4.1 | 9.7 |
| 2021 | 0.0 | 0.0 | 0.0 |  | 0.4 | 0.1 | 0.5 |  | 0.4 | 0.1 | 0.5 |
| 2022 | 0.0 | 0.0 | 0.0 |  | 3.8 | 3.0 | 6.7 |  | 3.7 | 3.0 | 6.7 |
| 2023 | 0.0 | 39.6 | 39.6 |  | 133.0 | 110.5 | 243.5 |  | 133.0 | 150.2 | 283.2 |
| 2024 | 0.0 | 0.0 | 0.0 |  | 171.5 | 130.5 | 302.0 |  | 171.5 | 130.5 | 302.0 |
| Total | 229.6 | 249.4 | 479.0 |  | 925.2 | 802.7 | 1727.9 |  | 1154.8 | 1052.1 | 2206.9 |

YLL years of life lost; YLD years lived with disability; DALY disability-adjusted life year.
